# Eastern Ohio Dental Association

**Published:** 1871-12

**Authors:** D. B. McLain

**Affiliations:** Secretary.


### ARTICLE V.

### *Eastern Ohio Dental Association.*

An adjourned meeting of a preliminary organization for the purpose of forming a Dental Association was held in Canton on Tuesday, August 29th, 1871, in Dr. J. H. Siddall's rooms.

Dr. Lyder, of Alliance, in the chair.

Dr. McLain, of New Lisbon, chairman of committee on constitution and by-laws, reported having procured a copy of the constitution and by-laws of the Northern Ohio Dental Association, which were adopted with some alterations; the name adopted being "Eastern Ohio Dental Association."

The following officers were elected for one year:

President, Dr. S. C. Whinery, of Salem; Vice President, Dr. J. W. Lyder, of Alliance; Secretary, Dr. D. B. McLain, of New Lisbon; Treasurer, Dr. A. J. Douds, of Canton; Examining Committee, Drs. J. H. Siddall, of Canton, J. M. Porter, Massillon, and C. M. Richmond, of Garrettsville.

Convention adjourned for dinner, and were hospitably entertained by Dr. Siddall, who evidently knows how to take care of the inner man.

After dinner the following dentists were present:

J. H. Siddall, Canton, T. J. Barclay, Columbiana, L. A. Hole, Salem, W. A. Porter, Dalton, J. M. Porter, Massillon, C. M. Richmond, Garrettsville, J. W. Lyder, Alliance, D. B. McLain, New Lisbon,—Craig, Canton.

There are no members belonging to the Association yet, as all must pass an examination and be recommended by the examining committee who report at next meeting the names only of those found worthy.

Dr. J. M. Porter read a very able paper on the pathology and treatment of alveolar abscess. The subject was then discussed by nearly all present.

The subject of filling with smooth pointed instruments was next discussed, Dr. J. M. Porter having performed a clinical operation of that nature in the forenoon.

The subject of specialties in dental practice was warmly discussed.

The following subjects were selected for discussion at the next meeting:

Anæsthesia, The Gums and their Diseases, and When is Extraction Indicated.

After passing a vote of thanks to Dr. Siddall for the use of his rooms and his turkey, the Society adjourned to meet in Alliance on the last Tuesday in February.

I omitted to state that Alliance is the permanent place of meeting, and the meetings are to be semi-annual.

D. B. McLain, Secretary.

---

### SELECTED ARTICLES.

